# Nanotheranostic Trojan Horse for visualization and photo-immunotherapy of multidrug-resistant bacterial infection

**DOI:** 10.1186/s12951-023-02267-6

**Published:** 2023-12-20

**Authors:** Xin Pang, Haohang Xu, Qishun Geng, Yu Han, Huiya Zhang, Heng Liu, Xiao Zhang, Mingsan Miao

**Affiliations:** 1 School of Pharmacy, Henan University of Traditional Chinese Medicine, Zhengzhou, 450046 China; 2 China-Japan Friendship Hospital (Institute of Clinical Medical Sciences), Chinese Academy of Medical Sciences & Peking Union Medical College, Beijing, 100193 China; 3 Joint Institute of Management and Science University, Henan University of Traditional Chinese Medicine, Zhengzhou, 450046 China; 4 Department of Radiology, PLA Rocket Force Characteristic Medical Center, Beijing, 100088 China; 5 Academy of Chinese Medical Sciences, Henan University of Traditional Chinese Medicine, Zhengzhou, 450046 China

**Keywords:** Antibacterial, Monocyte-hitchhiking, Photo-immunotherapy, Theranostics, Bacterial Infection

## Abstract

Effective diagnosis and therapy for bacterial infections, especially those caused by multidrug-resistant (MDR) species, greatly challenge current antimicrobial stewardship. Monocytes, which can chemotactically migrate from the blood to infection site and elicit a robust infection infiltration, provide a golden opportunity for bacterial theranostics. Here, a nano-Trojan Horse was facilely engineered using mannose-functionalized manganese-eumelanin coordination nanoparticles (denoted as MP-MENP) for precise two-step localization and potent photothermal-immunotherapy of MDR bacterial infection. Taking advantage of the selective recognition between mannose and inflammation-associated monocytes, the MP-MENP could be passively piggybacked to infection site by circulating monocytes, and also actively target infiltrated monocytes that are already accumulated in infection microenvironment. Such dual-pronged targeting enabled an efficient imaging diagnosis of bacterial infection. Upon laser irradiation, the MP-MENP robustly produced local hyperemia to ablate bacteria, both extracellularly and intracellularly. Further combined with photothermal therapy-induced immunogenic cell death and MP-MENP-mediated macrophage reprogramming, the immunosuppressive infection microenvironment was significantly relieved, allowing an enhanced antibacterial immunity. Collectively, the proposed nanotheranostic Trojan Horse, which integrates dual-pronged targeting, precise imaging diagnosis, and high-performance photothermal immunotherapy, promises a new way for complete eradication of MDR bacterial infection.

**Supplementary Information:**

The online version contains supplementary material available at 10.1186/s12951-023-02267-6.

## Introduction

Pathogenic bacterial infections, especially those caused by multidrug resistant (MDR) species, are an ever-growing global crisis in human medicine [1]. Complex infection phenotypes is one of the culprits for clinical failures. In addition to planktonic form, some pathogenic bacteria have evolved to invade and survive inside host phagocytes, creating an intracellular shelter from external drug attack [2]. Meanwhile, the immune-suppressive infection microenvironment greatly limits the establishment of robust antibacterial immunologic responses, resulting in an inability to clear invading pathogens [3]. Further suffering from undeveloped clinical infection detection, misdiagnosis and subsequent mismanagement continuously increase, also aggravating the antibacterial stewardship [4]. Plagued by drying antibiotic pipeline, alternative approaches to combat MDR bacteria, particularly to integrate broad-spectrum disinfection, immune restoration and imaging diagnosis, are urgently desired in this worldwide battle.

Physical (e.g., light, heat, ultrasound) therapeutics show great performance for a variety of diseases due to their non-invasiveness, spatiotemporal controllability, and low systemic toxicity [5]. As a representative paradigm, photothermal therapy (PTT), which ablates targets by local hyperthermia upon light stimulation, is highly potent for virtually all cells without resistance concern [6, 7]. It is a great bonus that most of photothermal agents possess inherent optical/magnetic properties and therefore can be used as imaging probes for disease diagnosis. More importantly, PTT has been widely reported to trigger immunogenic cell death (ICD) for the release of tumor-associated antigens and damage-associated molecular patterns (DAMPs), which can induce dendritic cell (DC) maturation and effector T cell infiltration, thereby alleviating the tumor immune suppression [8, 9]. Similarly, pathogenic bacteria as immunogenic cells are also expected to release multiple pathogen-associated molecular pattern molecules (PAMPs) and DMAPs under exogenous hyperthermia stress. These changes in PAMPs and DAMPs may elevate the levels of cytokines/chemokines produced by macrophages, activate the host immune cells, and elicit potent antibacterial immune responses [10]. Hence antibacterial photothermal therapy (aPTT) promises a new way for antibiotic-free theranostics to robustly combat MDR bacterial infections via combined photothermal-immunotherapy and in situ imaging diagnosis. However, the poor bacterial targeting of numerous photothermal agents poses a big challenge.

Endogenous cells are potentially powerful tools for delivering theranostic agents to specific infection targets. Immediately as the pathogenic bacteria invade, plenty of monocytes will be recruited from the bloodstream to the infected tissue by chemoattractant molecules liberated in the infection microenvironment [11]. Accompanied with the influx of monocytes during bacterial infection, the cell hitchhiking theranostic agents can serve as “Trojan Horses” to migrate orientedly towards the infection foci as part of the normal chemotaxis response, therefore making it available to accomplish spatial localization and rational treatment of bacterial infection [12]. Apart from blood monocytes, inflammation-associated monocytes which abundantly distribute in the infection microenvironment are also the potential target for bacterial theranostics. These infiltrated monocytes play an important role in pathogen phagocytosis, immunosuppression, and inflammatory response, and sometimes act as a shelter for bacteria colonization to induce chronic or recurrent infections [13–16]. As a result, dual-pronged targeting of such monocytes, both circulating and infiltrated types, may promise an outstanding strategy for bacterial theranostics with superior infection accumulation, potential immunomodulation, and increased sterilization especially for intracellular bacteria, but to date, the first example is still awaited.

**Fig. 1:**
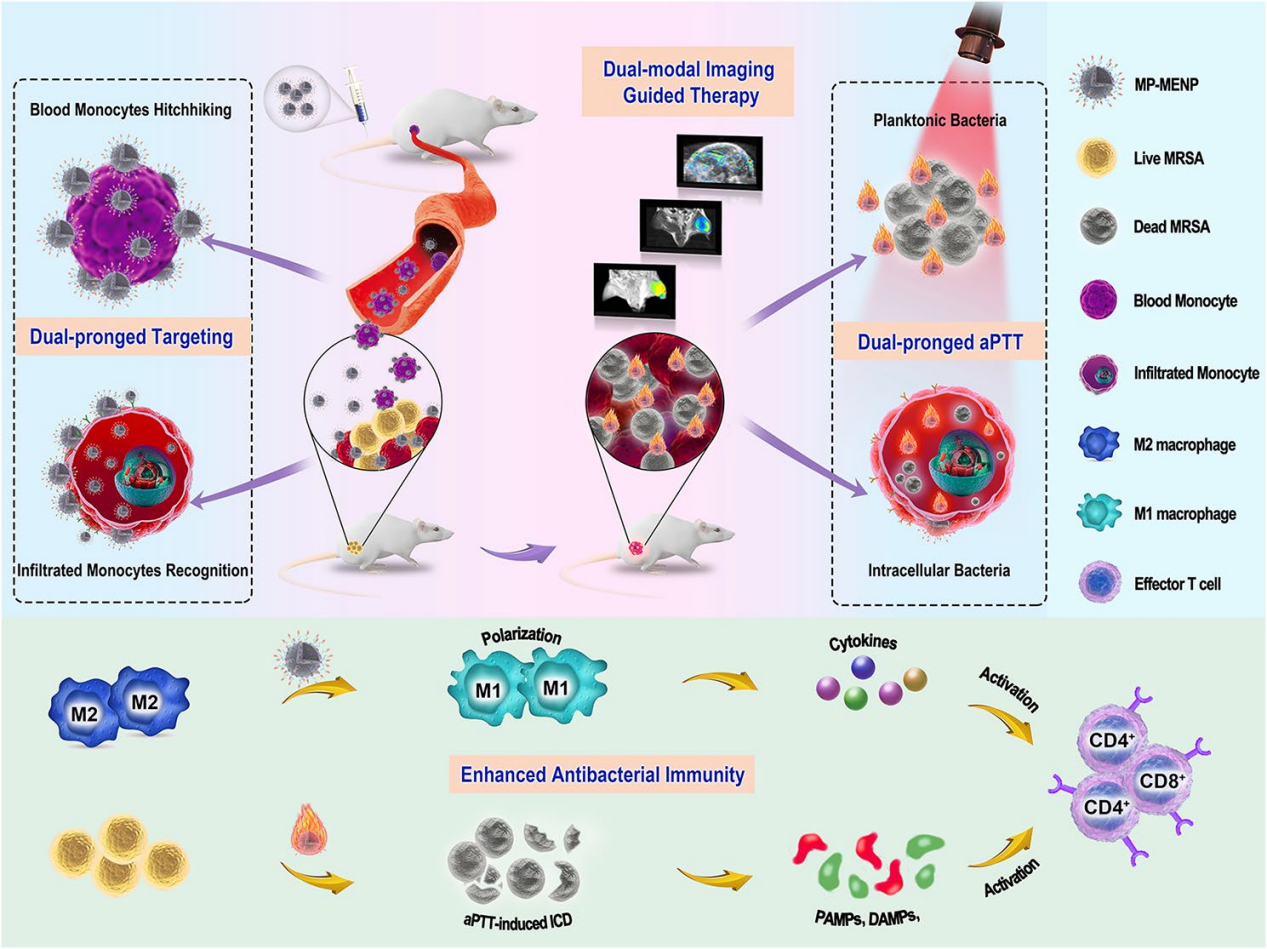
Schematic illustration of an endogenous cell hitchhiking nano-Trojan Horse (denoted as MP-MENP) for in situ visualization and photothermal-immunotherapy of MDR bacterial infection. By taking advantage of the selective recognition between mannose and inflammation-associated monocytes, the MP-MENP could be passively piggybacked to infection site by circulating monocytes, and also actively target infiltrated monocytes that are already accumulated in infection microenvironment. As a result, the bacterial infected area was selectively delineated by photoacoustic and magnetic resonance imaging, followed by a potent photothermal ablation for both planktonic and intracellular bacteria. Further combined with the synergistic effect of immunogenic cell death induced by photothermal therapy and macrophage reprogramming by MP-MENP, the antibacterial immunity of infection microenvironment was significantly enhanced, allowing a complete eradication of complicated MRSA infection

As a proof-of-concept, a nanotheranostic Trojan horse was developed using mannose-piloted manganese-eumelanin coordination nanoparticles (denoted as MP-MENP) for precise two-step localization and efficient photothermal-immunotherapy of complicated bacterial infection (Fig. 1). Benefiting from the potential biogenicity of eumelanin and manganese ions, such organic MP-MENP are expected to circumvent the poor biocompatibility/biodegradability issue, which usually troubles many inorganic photothermal materials such as gold nanorods, palladium nanosheets, and transitional metal dichalcogenides [17, 18]. More importantly, it has been reported that melanin can modulate macrophage via scavenging multiple reactive oxygen species (ROS) [19], therefore, the eumelanin-based MP-MENP are expected to restore the immunosuppressive bacterial infection microenvironment characterized by abnormally high ROS level. Due to the high expression of mannose receptor by monocytes/macrophages, the ligand-receptor recognition is assumed to take place not only in the circulation, resulting in monocytes piggybacking MP-MENP to infection sites, and also with the inflammatory macrophages that are already infiltrated in infection foci. Combined with the potential magnetic resonance imaging (MRI) and photoacoustic imaging (PAI) capabilities, such two-pronged targeting nanosystem will enable a superior accumulation and visualization of bacterial infection. Upon NIR stimulation, both planktonic MDR bacteria and intracellular methicillin-resistant *Staphylococcus aureus* (MRSA) can be photothermally ablated, inducing a higher level of infection-related ICD. The subsequent release of PAMPs and DAMPs expectantly provokes a series of immunological responses to create an immune-favorable infection microenvironment. Further combined with the great potential of MP-MENP in modulating macrophage repolarization and secretion of proinammatory cytokines, a large number of effector T cells are activated eventually, achieving an enhanced antibacterial immunity. Collectively, the proposed nanotheranostic Trojan Horse, by integrating dual-pronged targeting, precise imaging diagnosis, and high-performance photothermal immunotherapy, provides an intriguing direction for combating MDR bacterial infection.

## Experimental section

### Materials

3,4-dihydroxy-DL-phenylalanine (DL-DOPA), potassium permanganate (KMnO_4_), Mn ions standards, 3-(4,5-dimethylthiazol-2-yl)-2,5-diphenyltetrazolium bromide (MTT), were purchased from Aladdin Reagent (Los Angeles, Southern California, USA). Thiol-terminated mannose-poly (ethylene glycol) (mannose-PEG-SH) was obtained from Xi’an ruixi Biological Technologu Co. Ltd. All the chemicals and materials were used as received without further purification unless otherwise mentioned. Deionized (DI) water (Millipore Milli-Q grade, 18.2 MΩ) was used throughout the experiments. Clinically isolated extended-spectrum β-lactamase *Escherichia coli* (ESBL-producing *E. coli*), MDR *Klebsiella pneumoniae* (*K. pneumoniae*), MDR *Pseudomonas aeruginosa* (*P. aeruginosa*), and MDR *Bacillus* were kindly provided by Fuzhou General Hospital. Methicillin-resistant Staphylococcus aureus (MRSA) was collected from The First Affiliated Hospital, Sun Yat-sen University.

### Synthesis of MENP and MP-MENP

The MENP were synthesized based on our previous report with minor modifications. Briefly, DL-DOPA (60 mL, 10 mM) suspension was introduced into a 100-mL flask and heated to 50 °C. Then, KMnO_4_ solution (1.8 mL, 100 mM) after ultrasonic dispersion was rapidly added under vigorous stirring. This mixture was maintained at 50 °C for 6 h to form the MENP. To remove excess precursors and reactants, the obtained MENP were washed five times with deionized water via centrifugation (17,500 rpm, 15 min), and finally dispersed in water.

MP-MENP were synthesized by mixing MENP with mannose-PEG-SH at a feeding mass ratio of 1:5 in alkaline buffer solution (pH ≈ 10.0). After vigorous stirring overnight at room temperature, the products were washed several times using deionized water to remove residual mannose-PEG-SH. The final MP-MENP were dispersed in water and stored at 4 °C until use.

### Characterization of MP-MENP

The hydrodynamic diameter and ζ potential were detected by dynamic light scanning (DLS) (NanoZS 90, Malvern, USA). Transmission electron microscopy (TEM, Tecnai G2 Spirit BioTwin, FEI, USA) was used to observe the morphology of MP-MENP. The concentration of MP-MENP was measured using UV − vis − NIR spectroscopy (Cary 5000, Agilent, USA) at 808 nm wavelength. The Mn content was determined by inductively coupled plasma atomic emission spectroscopy (ICP-AES, Thermo) following digestion by aqua regia overnight.

### Photothermal effect of MP-MENP

In this study, 200 µL MP-MENP aqueous solution with different concentrations (0-200 µg/mL) was exposed to a 808 nm laser (2.0 W/cm^2^, 5 min), and the sample temperature was continually recorded by an infrared thermal camera. Deionized water was set as the control.

### Dual-modal imaging of MP-MENP

The PA signals of MP-MENP were collected using a preclinical PA imaging system (Endra Nexus 128, Ann Arbor, MI) upon 808 nm laser excitation with a pulse width of 7 ns and a repetition rate of 20 Hz. The moderate laser energy maintains at ∼5 mJ/cm^2^. The MRI relaxivity under various magnetic fields was determined by a 7.0 T small animal MR scanner (Bio-Spec, Bruker, Karlsruhe, Germany), a 3.0 T clinical scanner (Siemens, Prisma, Munich, Germany), and a 1.5 T HT-MICNMR-60 benchtop relaxometer (Huantong Corporation, Shanghai, China), respectively. The samples were dissolved in water containing 1% agarose in Eppendorf tubes (1.5 mL).

### In vitro anti-bacterial study on planktonic bacteria

Five clinically isolated bacterial strains, including MRSA, MDR *Bacillus*, ESBL-producing *E. coli*, MDR *K. pneumoniae*, and MDR *P. aeruginosa*, were employed in our experiment. The concentration of bacteria was measured by the optical density at 600 nm via UV − vis − NIR spectroscopy. To assess the antibacterial ability of MP-MENP, bacteria (10^6^ CFU/mL) were incubated with different concentrations of MP-MENP (6.25, 12.5, 25, 50 µg/mL). The bacteria without MP-MENP co-incubation was used as control. The bacteria suspensions were irradiated using a 808 nm laser (2 W/cm^2^, 5 min). Then, the samples were serially diluted. 100 µL of diluted bacterial suspensions was spread on lysogeny broth (LB) agar plates and cultured for 24 h to observe the number of bacterial colonies. The MP-MENP -treated bacteria with no laser irradiation were counted following the aforementioned procedures. Each sample was prepared in triplicate.

### Intracellular antibacterial activity

RAW264.7 murine macrophage cells were seeded on 96-well plates at a density of about 10^4^ cells/well and infected with MRSA at a ratio of 10–20 bacteria per macrophage. To eliminate the extracellular bacteria, 50 µg/ml gentamycin was added into culture media (DMEM). After overnight, the MRSA-infected macrophages were cultured in fresh medium with MENP or MP-MENP at a concentration of 100 µg/mL for 4 h. The group without nanoparticles treatment was used as control. Then, the cell culture medium was replaced with fresh medium, and the MRSA-infected macrophages were irradiated at 808 nm, 2 W/cm^2^ laser for 5 min. The intracellular bacterial viability was determined by lysing infected macrophages in distilled water and then plating the lysates on LB plates for culture and counting of bacterial colonies. The MRSA-infected cells without laser irradiation were counted following the aforementioned procedures. Each sample was prepared in triplicate.

For the dose-dependent antibacterial assay of MP-MENP, the MRSA-infected macrophage cells were incubated with MP-MENP at different final concentrations (12.5, 50, 100, 200 µg/mL). After 4 h incubation, the mixture was treated with or without laser irradiation. Then, the cells were lysed to isolate MRSA bacteria for colony counting. The cells without MP-MENP treatment were conducted as control.

### In vitro PA and MR imaging

The MP-MENP at different final concentrations (50, 100, 200 µg/mL) were incubated with RAW264.7 macrophage cells at 37 °C for 4 h. Subsequently, the cells were collected and resuspended in 1% agarose for imaging evaluation. The PA and MR images were recorded using a 7.0 T small animal MRI scanner and NEXUS 128 scanner, respectively. The signal intensity was determined through analyzing the region of interest.

### Membrane integrity measurement

The MRSA were cultured with 200 µg/mL MP-MENP. After laser irradiation, the bacteria were isolated and then stained by SYTO 9 and propidium iodide (PI) for 30 min in the dark. Afterward, bacteria were washed with saline for several times. Individual suspensions were immobilized on glass slides and imaged using laser scanning confocal microscope (Olympus, FV1200).

### Bacterial morphology study

The bacterial samples were prepared according to the study of Live/Dead Bacterial Staining Assay. After pretreatment, the bacteria were fixed using paraformaldehyde for 4 h and observed using scanning electron microscope (SEM).

### Western blotting

Equal volumes of MRSA from different groups were collected and washed twice with PBS. After ultrasonication, bacterial sediments were removed by centrifugation. The supernatant was incubated with 1% Protease Inhibitor Cocktail and then mixed with a loading buffer followed by heating at 100 °C for 10 min. Proteins from different groups were added to SDS-PAGE and subsequently transferred to the poly(vinylidene difluoride) membrane. After being blocked, the membrane was consecutively incubated with antibodies against DnaK (1:1000 dilution) and GAPDH (1:10,000 dilution). The bands were washed for HRP-conjugated secondary antibodies incubation. Finally, the proteins were imaged, and the chemiluminescence signals were detected.

### Macrophage repolarization

RAW264.7 cells were first pre-stimulated with 100 ng/mL of IL-4 for 24 h to polarize them into M2-type macrophages, and then co-incubated with LPS, MENP, or MP- MENP for another 24 h. The cell supernatant was carefully collected for the cytokine secretion assay using ELISA assay kits. Meanwhile, the cells were collected for APC-conjugated anti-mouse CD86 antibody (Biolegend, USA) and 0.5 µg PE-conjugated anti-mouse CD206 antibody (Biolegend, USA) incubation, and then detected using flow cytometer.

### Migration activity of RAW264.7

The migration activity of RAW264.7 was confirmed using a transwell assay. Briefly, RAW264.7 cells (1 × 10^5^ cells/sample) were seeded onto 8 μm transwells. Next, 2.5 mg/mL of MENP/MP-MENP were added into the lower chambers, while LPS and PBS were also added into the other two groups as positive and negative controls. Following 24 h incubation, cells on the bottom of the transwell were stained with crystal violet and counted using optical microscopy.

### Phagocytic activity of RAW264.7

RAW264.7 cells (2 × 10^5^ cells/well) were seeded onto a 6-well plate and treated with PBS, LPS, MENP and MP-MENP groups, respectively. The assay for gentamicin protection was then carried out. After 24 h co-incubation, the supernatant was replaced with fresh DMEM containing MRSA (10^7^ CFU/mL) for 30 min incubation. Then, the suspension was removed and new DMEM containing 200 µg/mL of gentamicin was added. Following 1 h culture to eradicate the extracellular bacteria, cells were washed with PBS thoroughly. After that, 1 mL of 1% Triton X-100 was added to lyse macrophages, thereby releasing intracellular bacteria. Finally, the Triton X solution from each well was collected to calculate the bacteria by gradient dilution and plate counting methods.

### Animal studies

Male Balb/c mice (6 weeks, ~ 20 g) were purchased from Zhengzhou Hua-xing Laboratory Animal Center. All animal studies were carried out in compliance with the protocols approved by the Animal Management and Ethics Committee of Henan University of Traditional Chinese Medicine. To assess the theranostic effect of MP-MENP, a mice model of bacterial infection was established by subcutaneous injection of MRSA bacteria (10^8^ CFU/mL, 100 µL) into the right thigh of mouse, while saline was injected to the left thigh as a control. After 24 h infection the mice were randomly divided into several groups (n = 5) for further use.

### In vivo imaging of MRSA infection

The MRSA-infected mice were injected with MENP or MP-MENP (at a dose of 20 mg/kg) via the tail vein, respectively. In the case of competitive inhibition experiment, the infected sites were in situ injected with mannose to block the infiltrated monocytes before administrating MP-MENP. The PA images from the MRSA-infected region at 0, 1, 3, 6, 12, and 24 h were captured by FUJIFILM visualsonics (Toronto, Canada). The *T*_*1*_- and *T*_*2*_- weighted MR images containing coronal planes were acquired prior to and at 6 h postinjection of MP-MENP using a 3.0 T clinical scanner (Siemens, Prisma, Munich, Germany). The contrast enhancement was quantified by analyzing the ROI.

### In vivo PTT of MRSA infection

To evaluate antibacterial PTT efficacy of MP-MENP in vivo, mice with MRSA infection were randomly divided into four groups (n = 5): (1) saline without any treatment, (2) saline with 808 nm laser irradiation (2 W/cm^2^, 5 min), (3) MP-MENP alone, and (4) MP-MENP with 808 nm laser irradiation (2 W/cm^2^, 5 min). The laser exposure was carried out at 6 h post-injection. The infected areas were recorded every 3 days. After 12 days of monitoring period, the mice were sacrificed, and the infected tissues were harvested for hematoxylin and eosin (H&E) staining and CFU analysis.

### In vivo immunomodulation

After each treatment on day 2, mice were sacrificed and the infected skin tissues were collected for immunofluorescence (IF) labeled with F4/80, CD86, CD206 related antibodies. Meanwhile, IF staining of HSP70, CD4^+^ T cells, and CD8^+^ T cells also carried out to verify the immunological effects. Moreover, the collected skin tissues were also quickly frozen in liquid nitrogen on day 2, and then sent to Novogene Co. Ltd. (China) for high-throughput sequencing analysis. The enzyme-linked immunosorbent assay (ELISA) was introduced to measure the serum cytokines according to the protocols recommended by manufacturer (ExCell Bio, Shanghai, China).

### Safety evaluation

For cytotoxicity assay, human umbilical vein endothelial cells (HUVEC), RAW264.7 murine macrophage cells, and human hepatic cells LO2 were used. The cells were cultured overnight on 96-well plates (10 000 cells/well), and then co-incubated with MP-MENP at different final concentrations (0, 50, 100, and 200 µg/mL) for 24 h. Subsequently, the cells were washed three times using PBS. The MTT solution (0.5 mg/mL, 10 µL) was added to each well. After 4 h incubation, the cell culture medium was replaced by 150 µL DMSO. The optical density of each well was determined at 490 nm using a microplate reader.

Hemolysis analysis of MP-MENP was tested on red blood cells (RBCs). The erythrocytes were collected through centrifugation (1500 rpm, 15 min), and then washed 5 times with saline. The centrifuged erythrocytes (3 mL) were mixed with saline (11 mL) to obtain the stock dispersion of RBCs. Then, 100 µL stock dispersion was added to saline solution (0.9 mL) containing MP-MENP at different concentrations. The final RBCs level was about 4%. Hereinto, deionized water and saline were conducted as the positive and negative controls, respectively. After 3 h incubation at 37 ℃, the samples were centrifuged at 12,000 rpm/min for 15 min. The percentage of hemolysis was determined by UV − vis analysis of the supernatant at 540 nm absorbance, and calculated using the following formula:

Percent hemolysis = ((A_S_ – A_N_) / (A_P_ – A_N_)) ×100%

A_S_ represents the absorbance of MP-MENP in RBCs suspension, A_N_ represents the absorbance of RBCs suspension with saline treatment, and A_P_ is the absorbance of sample treated with deionized water.

For in vivo safety evaluation, healthy Balb/c mice (6 weeks, ~ 20 g) were randomly divided into two groups (n = 5) for saline or MP-MENP (20 mg/kg) administration. After 1-day postinjection, the mice were sacrificed. The major organs including heart, liver, spleen, lung, and kidney were collected and fixed using 4% paraformaldehyde solution for H&E staining.

### Statistical analysis

Data analysis was conducted with the GraphPad Prism software. All data in this work were presented as mean ± SD. Statistical significance was determined by a One-way analysis of variance (ANOVA). * means P < 0.05, ** means P < 0.01, and *** means P < 0.001.

#### Result and discussion

##### Characterization of MP-MENP

**Fig. 2:**
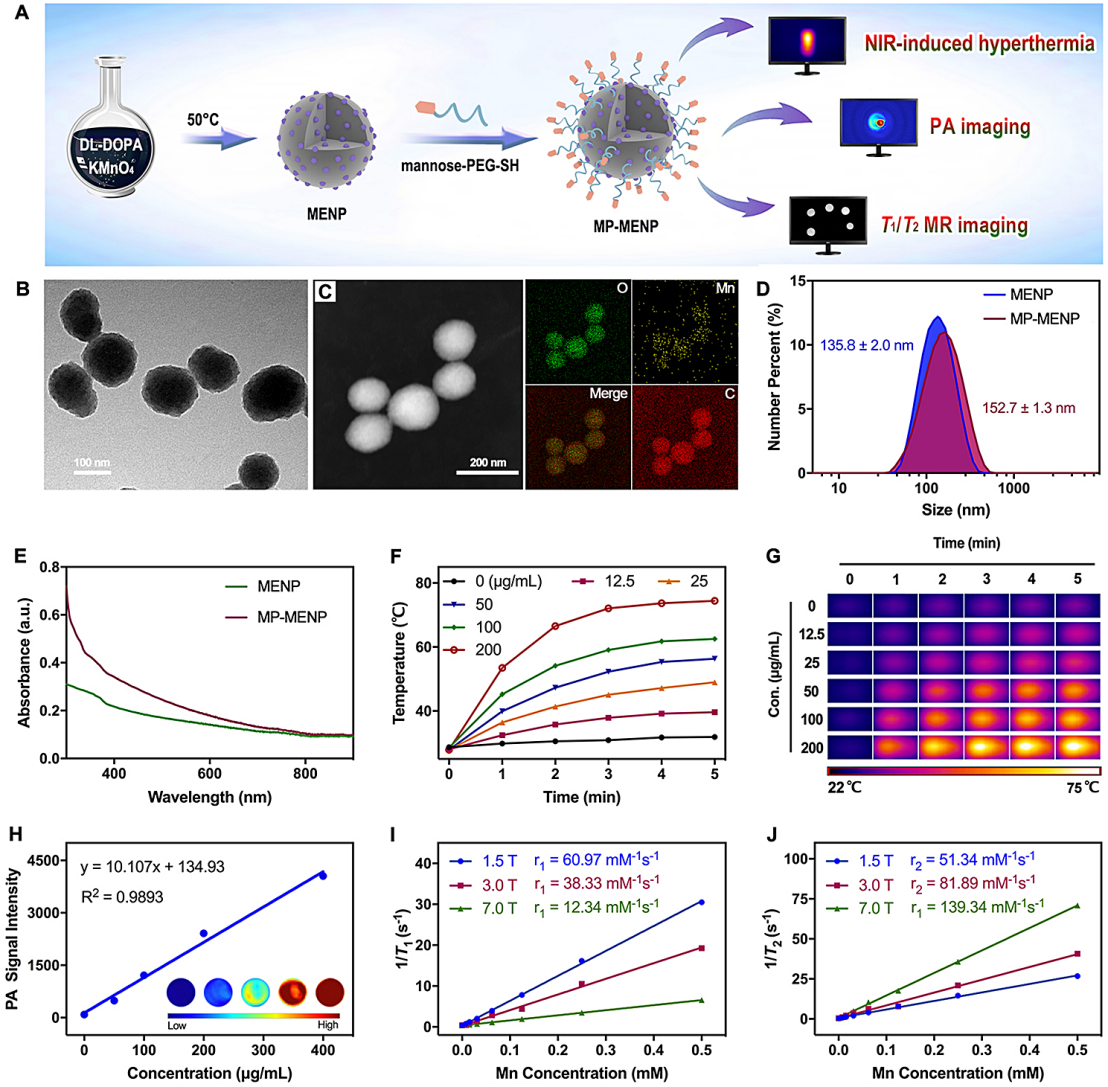
(**A**) Schematic illustration of the preperation and functionality of MP-MENP. (**B**) TEM image and (**C**) HAADF-STEM-EDX mapping of MP-MENP. (**D**) Size distribution and (**E**) UV-vis-NIR − vis spectra of MENP or MP-MENP in deionized water. (**F**) The temperature variation and (**G**) thermographic images of MP-MENP upon 808 nm laser irradiation (2 W/cm^2^, 5 min). (**H**) PA imaging signals of MP-MENP with different concentrations. The inset shows the corresponding PA images of MP-MENP. The linear relationship for the (**I**) *r*_1_ and (**J**) *r*_2_ relaxivities of MP-MENP as a function of Mn concentration

We first fabricated water-dispersible manganese-eumelanin coordination nanoparticles (MENP) through a one-pot intrapolymerization doping (IPD) strategy (Fig. 2A). After simple chemical oxidation-polymerization of the DL-DOPA precursor with KMnO_4_, MENP were successfully obtained with a Mn loading efficiency of 8.2% wt/wt. Subsequently, mannose-terminal PEG was introduced to modify the pristine MENPs for macrophage targeting. Observed by transmission electron microscopy (TEM), the mannose-decorated MENPs (defined as MP-MENP) had a well-controlled spherical morphology (Fig. 2B). The presence of oxygen, carbon, and manganese in MP-MENP was revealed by the high­angle annular dark field scanning transmission electron microscopy energy dispersive X-ray spectroscopy (HAADF-STEM­EDX) mapping. The average diameter of MP-MENP was about 152.7 nm as shown by dynamic light scattering (Fig. 2D), which is slightly larger than pristine MENPs. The surface zeta potentials of MENPs and MP-MENP were − 30.7 and − 35.8 mV, respectively. The differences of size and zeta potential between MENP and MP-MENP may be attributed to the presence of hydration shell formed between abundant hydroxyl groups in mannose-terminal PEG and surrounding water molecules, which can potentially stabilize the nanoparticles as well as change their apparent properties.

#### Photothermal and imaging effects of MP-MENP

Photothermal performance is the key factor dominating the efficiency of antibacterial effect and subsequent immune activation. From the UV-vis-NIR absorption spectrum analysis (Fig. 2E), there was no obvious NIR absorption change between MENP and MP-MENP in the NIR region, indicating that the potential photothermal property of nanomaterials will not be impaired by mannose PEGylation. Figure 2F shows a gradual rise of temperature signals with increasing MP-MENP exposed to laser irradiation (808 nm). As the irradiation time prolonged, the temperature of MP-MENP underwent two phases (i.e., a steady rise and leveling off) in succession (Fig. 2F, G). When arriving at steady-state, the temperature of MP-MENP aqueous solution could stay above 50 °C, even though its concentration is low as 25 µg/mL. Such excellent photothermal effect is highly conducive to further biological application. We then investigated the capability of MP-MENP as a PAI and MRI contrast agent. As expected, the PA signals of MP-MENP presented a gradual enhancement as the concentration increased (Fig. 2H). Similarly, a prominent concentration-dependent contrast enhancement was observed in both *T*_1_WI and *T*_2_WI., and the relaxivity variations at different magnetic fields (from 1.5 to 7.0 T) induced an obvious increase in the ratio of longitudinal (*r*_1_) to transverse (*r*_2_) relaxivity (Fig. 2I, J). All of these suggested that the MP-MENP could serve as a potential nanoplatform for imaging visualization and photothermal ablation of bacterial infection.

#### In vitro antibacterial activity on planktonic strains

**Fig. 3:**
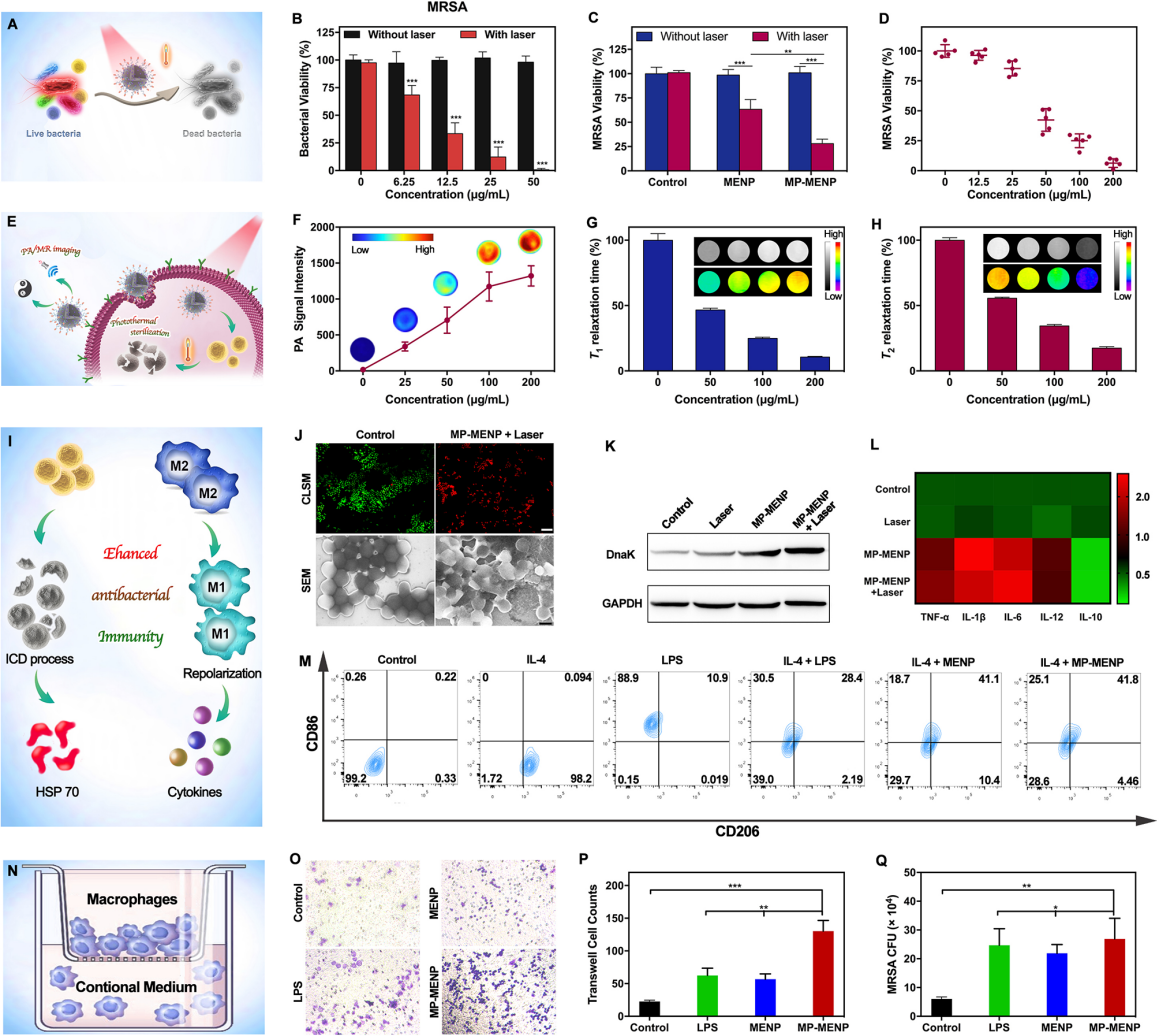
(**A**) Schematic illustration of MP-MENP mediated PTT against planktonic bacteria. The bacterial viability of (**B**) MRSA *versus* the MP-MENP concentrations with/without laser irradiation (808 nm, 2 W/cm^2^, 5 min). (**C**) The bacteria viability of MRSA with or without PTT by saline, MENP, and MP-MENP. (**D**) Concentration-dependence of MRSA survival with MP-MENP upon laser irradiation (808 nm, 2 W/cm^2^, 5 min). (**E**) Schematic illustration of the theranostic MP-MENP for intracellular MRSA. (**F**) PA signals, normalized (**G**) *T*_*1*_ and (**H**) *T*_*2*_ relaxation times of MRSA-infected macrophages after co-incubation with MP-MENP. The insets in panels (**F**), (**G**), and (**H**) show corresponding PA, *T*_*1*_WI, and *T*_*2*_WI images, respectively. (**I**) Schematic illustration of immuno-modulation effect by MP-MENP. (**J**) Representative overlapping fluorescence images for a live/dead bacterial staining assay (sacle bar: 10 μm) and SEM images (sacle bar: 500 nm) of MRSA with or without MP-MENP-mediated PTT. (**K**) Western blot of HSP70 expression by RAW264.7 macrophages with different treatments. (**L**) ELISA results of cytokines secreted by RAW264.7 after different treatments. (**M**) Detection of macrophage surface markers CD86 (M1 macrophage marker) and CD206 (M2 macrophage marker) using a flow cytometer. (**N**) Schematic illustration of the transwell co-culture system. (**O**) Typical images of RAW264.7 cells cultured on transwell stimulated with different conditional media. (**P**) Cell count results of the migrated RAW264.7 on the bottom of the upper chamber of transwells in different conditions. (**Q**) Counting results of phagocytized MRSA by RAW264.7 treated in different groups. * means P < 0.05, ** means P < 0.01, and *** means P < 0.001

For antibiotic-resistant bacteria, locally increasing temperature (50 °C) by photothermal agents will cause a great inhibition on microbial metabolism, culminating in cell death when extensive enough [20]. To evaluate the antibacterial photothermal activity of MP-MENP, five clinically isolated MDR strains were selected (Fig. 3A). Among them, MRSA and MDR *Bacillus* are classified as Gram-positive bacteria, and three other strains, including ESBL E. coli, MDR *K. pneumoniae* and MDR *P. aeruginosa*, are the well-known Gram-negative species. The killing or antibacterial efficacy of MP-MENP at different concentrations was assessed by their corresponding bacterial viability using colony counting method. As shown in Fig. 3B and S1, the viability values were above 90% for all bacterial strains in the absence of NIR irradiation, indicating an almost negligible antibacterial efficacy of the MP-MENP itself. By contrast, the groups containing MP-MENP and NIR exposure showed obvious growth inhibition, and the bacterial viability gradually decreased to less than 5% with the increase of MP-MENP concentration to 50 µg/mL. The influence of NIR light could be also excluded as no significant differences on the viability were found at 0 µg/mL between the above two tests. Strikingly, the photothermal toxicity of MP-MENP was broadly effective in both Gram-positive bacteria and Gram-positive species. It is generally known that treating infections caused by MDR organisms, particularly their Gram-negative species, is a significant challenge for medical practitioners and greatly increases patient mortality and cost of care globally. Considering the broad-spectrum photothermal damage, our developed MP-MENP may provide a novel-acting approach for sterilization regardless of bacterial species and drug-resistance.

#### In vitro antibacterial activity on intracellular MRSA

Intracellular infections are an impermeable barrier hindering the effective management of bacterial theranostics. When infection does occur, monocytes not only can chemotactically migrate to phagocytose invading organisms, but also serve as a potential shelter for bacteria, protecting them from attacks by antibacterial drugs. Once internalized by migratory monocytes, pathogens can survive within cells for extended periods and may reach distant sites to induce infection, which could result in long-term chronic or recurrent cases [21, 22]. In our study, the MP-MENP were functionalized with mannose which can target monocytes and then induce receptor mediated endocytosis. As a result, the bacteria hiding inside monocytes are expectantly eliminated by MP-MENP-mediated PTT. To verify this hypothesis, RAW 264.7 was selected as the model monocyte host for MRSA invasion. The surviving intracellular bacteria after different treatments were isolated and examined using colony counting method. As noted, NIR irradiation alone did not cause any viability inhibition on intracellular MRSA, while the bacteria treated with MENP showed remarkable growth inhibition upon NIR activation (Fig. 3C). Taking advantage of macrophage-targeting ability, the mannose-modified MP-MENP performed the most efficient photothermal inactivation of intracellular MRSA, and such antibacterial efficacy was concentration-dependent (Fig. 3D). Virtually all the intracellular MRSA were killed at the MP-MENP concentration of 200 µg/mL.

### In vitro imaging of monocytes by MP-MENP

To confirm that MP-MENP could successfully lighten macrophages, the RAW 264.7 macrophage cells were incubated with MP-MENP for 4 h, and then collected for PAI and MRI investigation (Fig. 3E). Cellular PA images demonstrated incubation concentration-dependent signal enhancement (Fig. 3F). The harvested macrophages also showed obvious positive and negative contrast enhancement on *T*_1_WI and *T*_2_WI, respectively. Both the *T*_1_ and *T*_2_ relaxation time of cells were gradually reduced as the incubation concentration of MP-MENP increase (Fig. 3G, H). These results elucidated that MP-MENP could be effectively internalized by macrophages and then implement the dual-modal imaging of the cells. Further coupled with potent photothermal ablation effect, MP-MENP expectantly act as a promising nanotheranostic to visualize infection and implement a broad-spectrum killing of both planktonic bacteria and intracellular strains.

### In vitro immunomodulation effect of MP-MENP

To verify the proposed immunomodulation route of MP-MENP (Fig. 3I**)**, SYTO 9 and PI co-staining was firstly introduced, in which the green fluorescent SYTO 9 was used to label live bacteria, and red dye of PI permeates only the damaged bacterial cell membrane and has been proposed as a common marker to investigate membrane integrity. As shown in Fig. 3J and S2, most of MRSA in control group presented green color *via* SYTO 9 staining, while the red PI fluorescence from damaged bacteria was widely found in MP-MENP-mediated PTT group. Further observed by SEM, the untreated MRSA bacteria had integral and smooth bodies (Fig. 3J). After exposure to MP-MENP and NIR laser, the bacterial morphology was dramatically changed, suggesting a robust bacterial disruption by MP-MENP-mediated PTT. The wrinkled, collapsed and even lysed cell walls will lead to the leakage of intracellular content for bacteria. Along with dead bacteria and debris, such PAMPs are promising to activate potential immune response [23, 24]. Considering the hyperthermia-induced bactericidal effect of MP-MENP, we then investigated the expression of heat-shock proteins (HSPs) by MRSA after PTT, because HSPs are representative of defense-related proteins that resist thermal stress. As danger signals, they are commonly found in almost all organisms, from bacteria to humans, and can be rapidly produced and released when cells suffer from elevated environmental temperatures [25, 26]. From Fig. 3K, the DnaK (a classical HSP70 homologue of *Staphylococcus aureus*) was significantly upregulated in bacteria treated with MP-MENP plus laser irradiation, which means an increased expression of HSP70. As a well-known ICD-related DAMPs, HSP70 has been widely reported with an ability to trigger immune response. Therefore, the upregulation of HSP70 expression after MP-MENP-mediated PTT shows great promise in enhancing antibacterial immunity.

The levels of inflammatory cytokines following different treatments in RAW 264.7cells were further measured by ELISA kit. As shown in Fig. 3L, the MP-MENP could significantly augment the secretion of M1-associated pro-inflammatory cytokines, including tumor necrosis factor (TNF)-α, interleukin (IL)-1β, interleukin (IL)-6, and interleukin (IL)-12. In contrast, the M2-associated anti-inflammatory cytokine of interleukin (IL)-10 was obviously downregulated by MP-MENP stimulation. In the case of bacterial infection, the immunosuppressive microenvironment will cause a switch of host infection-associated macrophages, from pro-inflammatory macrophages (M1) to anti-inflammatory macrophages (M2) in order to evade immunologic eradication. The M1-type macrophages could not only phagocyte and kill pathogenic bacteria directly, but also secret inflammatory factors to induce DCs maturation and thus reverse immune suppression [27, 28]. As a result, repolarizing macrophages into M1 phenotype is considered as a promising therapeutic strategy for bacterial infection. To evaluate the repolarization capability of MP-MENP on macrophages, IL4-was used to stimulate RAW264.7 macrophages (M2), and lipopolysaccharide (LPS) was introduced as a positive control to repolarize M2 macrophages into the classic inflammatory M1 population. The proportions of CD86 (M1 marker) and CD206 (M2 marker) macrophages were assessed by flow cytometry. Figure 3M revealed that the proportion of M1-type macrophages increased greatly (25.1%) in the IL-4 + MP-MENP group compared with the PBS alone (0.26%) and IL-4 group (0%). Moreover, there is few differences between the MENP and MP-MENP groups, which can be ascribed to the enhanced macrophage targeting and internalization provided by mannose modification. Such M1-type polarization and pro-inflammatory cytokines secretion are conducive to reactivate antibacterial functionality of macrophages, thereby reversing the immune microenvironment suppressed by pathogenic bacteria.

We then investigated the in vitro migration of macrophages using the transwell assay (Fig. 3N). It was observed that RAW264.7 cell migration on transwell was significantly enhanced in the presence of MP-MENP, which would allow macrophages to move rapidly to the infection site (Fig. 3O, P). To further evaluate the influence of nanoparticles on the anti-bacterial phagocytic capacity of macrophages, RAW264.7 cells after different treatments were co-cultured with MRSA. As shown in Fig. 3Q, the phagocytic number of MRSA in the MENP group was much more than that in the PBS group, and the modification of mannose further promoted the phagocytosis of macrophages. Collectively, MP-MENP was beneficial to reactivate antibacterial functions of macrophages, thus alleviating the immunosuppressive microenvironment of bacterial infection.

## In vivo diagnosis of MRSA infection

**Fig. 4:**
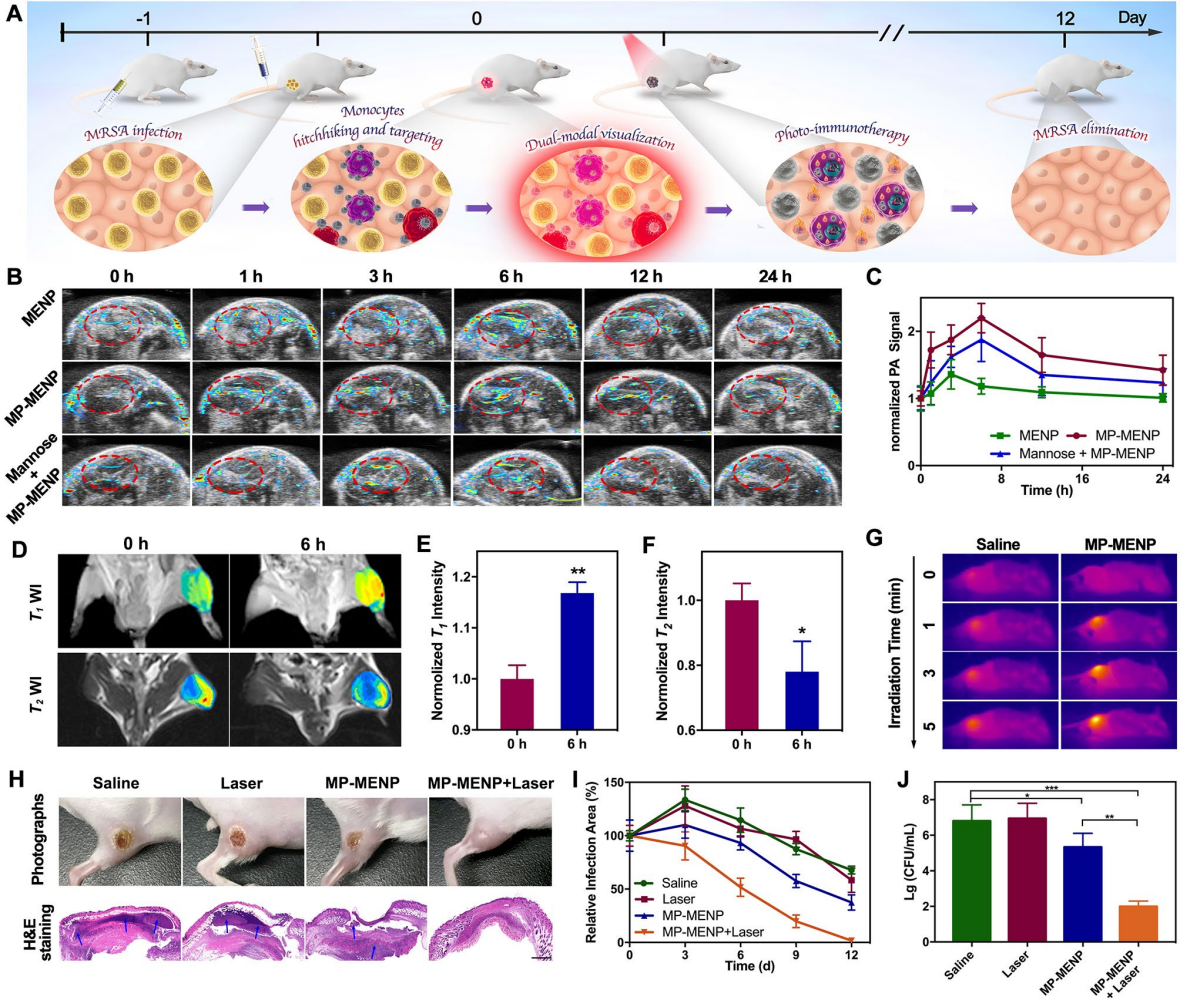
(**A**) The schedule of MRSA infection, systemic administration of MP-MENP, imaging diagnosis, and photo-immune eradiaction of infection in vivo. (**B**) PA images and (**C**) corresponding PA signal intensity of MRSA-infected area (red circles) from mice treated with MENP, MP-MENP, or mannose + MP-MENP. (**D**) *T*_*1*_-weighted and *T*_*2*_-weighted MR images and corresponding normalized (**E**) *T*_*1*_-weighted and (**F**) *T*_*2*_-weighted MR signal intensities of infected area in mice before and 6 h after i.v. injection with MP-MENP. (**G**) Thermal images of the infection regions upon laser irradition (808 nm 2 W/cm^2^, 5 min). (**H**) Representative digital photos and histological images of MRSA-infected region from mice with different treatments. The arrows in histological images indicate the inflammatory exudate and necrotic tissue. Scale bar: 40 μm. (**I**) Corresponding changes of wound areas. (**J**) The number of surviving MRSA on day 12 after treatment. * means P < 0.05, ** means P < 0.01, and *** means P < 0.001

Effective diagnosis of bacterial infection is imperative to guide therapeutic regimen and control the propagation of this disease. As one of the most notorious pathogenic bacteria, MRSA have evolved to cause complicated infections, always accompanied with extracellular and intracellular localization [29]. This significantly challenges the clinical diagnosis and therapy. Considering the unique imaging and chemotactic navigation properties, the MP-MENP are expected as a powerful dual-modal contrast agent to reveal the sites of MRSA infection (Fig. 4A). In this study, an infected mouse model was constructed by subcutaneous injection of MRSA bacteria (10^8^ CFU/mL, 100 µL) into the right thigh of the mouse, while the left thigh was injected with saline as a control. After intravenous administration, the diagnostic performance of MP-MENP was visualized and compared with MENP at different time points. Figure 4B shows that MENP was slightly accumulated to the infection site, and achieved the maximal accumulation at 3 h post-injection. By contrast, after modifying with mannose, the MP-MENP exhibited higher and more persistent PA emission at infection site. At 1 h post-injection, the infection area was easily distinguished from surrounding tissues due to its strong PA signal, and such specific accumulation was maintained until 12 h post-injection. To support our hypothesis that MP-MENP do hitchhike to monocytes upon intravenous injection, a competitive inhibition experiment was conducted. Before administrating MP-MENP, the infected sites of mice were in situ injected with mannose to block the infiltrated monocytes. Semiquantitative PA analysis revealed that infection areas pre-treated with mannose showed weak photoacoustic signals compared with MP-MENP group, confirming the potential targeting effect of MP-MENP to local macrophages in infection sites (Fig. 4C). Strikingly, the photoacoustic signals from the mannose + MP-MENP group were stronger than that from the MENP group. This indicates that MP-MENP implement a monocytes hitchhiking from the blood to infected locations and therefore achieve an increased infection accumulation. Further MRI studies of MRSA-infected mice also revealed similar results (Fig. 4D-F). At 6 h post-injection, the MP-MENP provided obviously positive and negative contrast enhancement on *T*_*1*_WI and *T*_*2*_WI at infection region, respectively. This clear PA and MR visualization of bacterial infection in vivo was mainly attributed to potent synergism of mannose-mediated monocytes hitchhike and the targeting to macrophages which abundantly infiltrate at infection sites. In addition, the potential multivalent targeting of mannose to bacteria may also play a part here [30, 31]. Compared to common-used golden standard in clinical infection diagnosis, such as tissue biopsies and cultures, our developed MP-MENP with real-time, non-invasive, and dual-modal imaging functions provides a promising diagnostic platform for bacterial infection.

### In vivo photothermal ablation therapy in MRSA infected mice

In vivo photothermal eradication of bacterial infection was subsequently studied in a mice model with MRSA-induced subcutaneous infection. The PTT efficacy of MP-MENP was assessed at 6 h postinjection upon laser exposure (808 nm, 2 W/cm^2^, 5 min). Figure 4G shows the thermal images of mice treated with saline or MP-MENP upon laser irradiation. The temperature of the infection area in MP-MENP + laser group rapidly increased and reached 56.7 °C after 5 min irradiation (Figure S3), which is higher that the antibacterial threshold (i.e. 50 °C). However, the maximum temperature was only 39.8 °C for the saline group, indicating that the hyperthermal effect in MP-MENP + Laser group was attributed to the highly accumulated MP-MENP, instead of laser alone. After the treatment, the areas of infection were measured every 3 days (Figure S4). In comparison with the groups of saline, laser alone or MP-MENP alone, the mice treated with MP-MENP plus laser irradiation exhibited the fastest healing rate. For them, the swelling and ulceration on the skin gradually disappeared, and a brand new intact epithelialization was found at day 12 (Fig. 4H, I). By contrast, inflammation and obvious abscess with scab on the mice skin remained in the other three groups. To further verify the PTT efficacy, we examined histological characteristics, residual MRSA bacterial number of the infection region after 12 days. H&E staining results indicated that the skin biopsy section in MP-MENP + Laser group presented normal morphological features with new capillaries and hair follicles, while the mice skins in other three groups suffered from massive inflammatory cell infiltration, local skin necrosis, and neutrophil accumulation (Fig. 4H). Moreover, the significantly reduced MRSA colonies in MP-MENP + Laser group also implied the great photothermal antibacterial efficacy of MP-MENP (Fig. 4J). Considering the highly potent therapeutic outcomes, the internalized MP-MENP may exert a photothermal ablation for intracellular bacteria, while the extracellular MP-MENP delivered by monocytes hitchhiking are expectantly more effective to planktonic bacteria. Such two-pronged antibacterial strategy facilitates the complete rescue of mice from MRSA infection.

### In vivo immuno-modulation of bacterial infection microenvironment

**Fig. 5:**
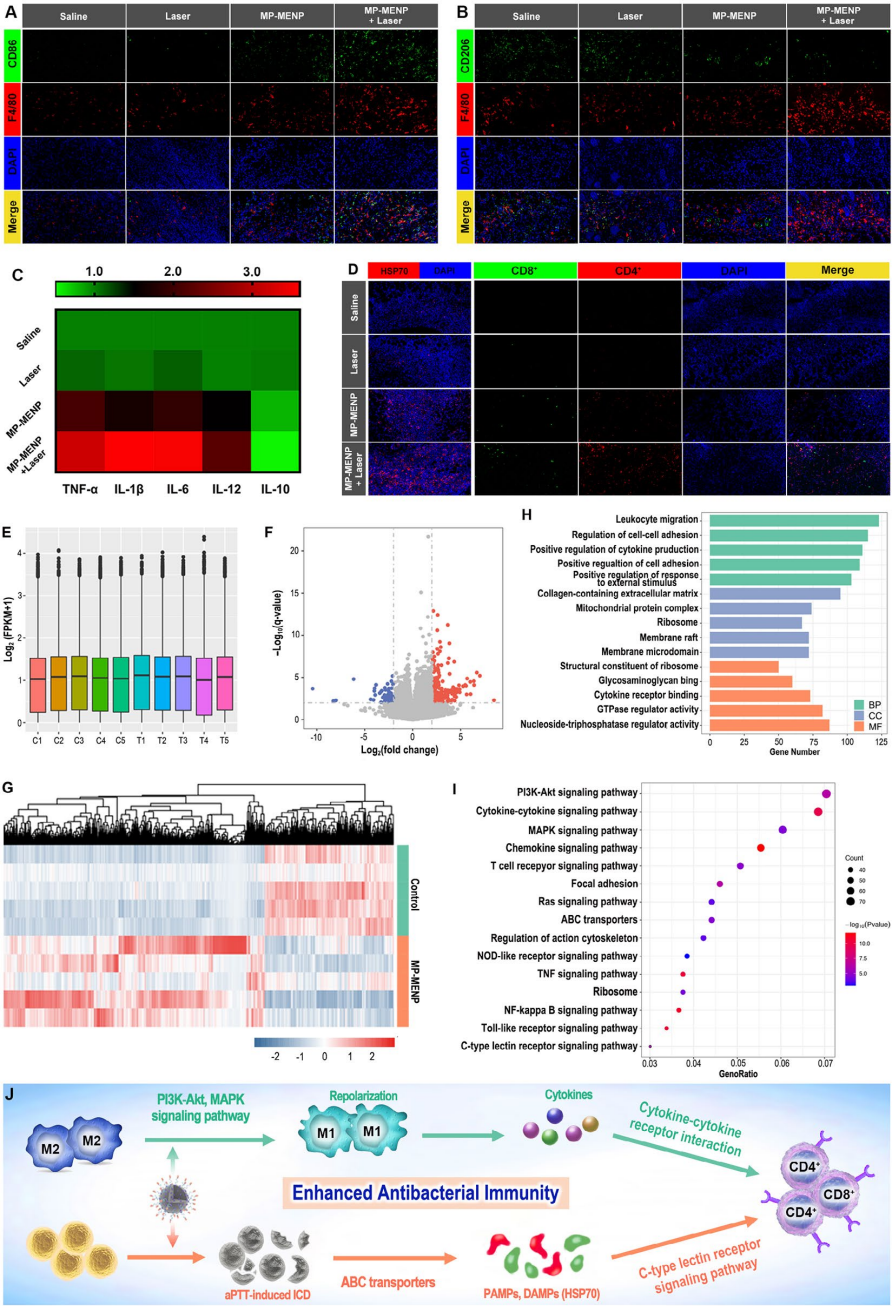
(**A**) The schedule of immuno-modulation process by MP-MENP. (**B**, **C**) IF results (F4/80^+^/CD86^+^: M1-type cell; F4/80^+^/CD206^+^: M2-type cell) of infected skin treated in different groups on days 2. (**D**) ELISA results of cytokines secreted by MRSA-infected tissues after different treatments. (**E**) IF results of HSP70, CD4^+^, and CD8^+^ T cells in infection microenvironment. (**F**) Transcriptomics analysis of the distribution of gene expression in control group (C1-C5)and MP-MENP mediated photothermal-immunotherapy group (T1-T5). (**G**) The volcano plots of the control vs. MP-MENP mediated photothermal-immunotherapy group. (**H**) The heat map of the reversed genes with significant differences. GO (**I**) and KEGG (**J**) enrichment analysis of the reversed genes. Nanotheranostic Trojan Horse for Visualization and Photo-Immunotherapy of Multidrug-Resistant Bacterial Infection

To gain insight into the effect of MP-MENP-mediated PTT on immune response, IF staining on day 2 was applied to evaluate the enrichment and polarization of macrophages. As shown in Fig. 5A and B and S5, more M1-type macrophages (red fluorescence) were present in subcutaneous tissues for MP-MENP and MP-MENP + Laser groups, implying that MP-MENP could act as a promising immune modulator to promote the aggregation and polarization of macrophages into M1 phenotype. When compared to the saline and laser groups, MP-MENP group presented more F4/80 fluorescence signals, indicating a higher infiltration of macrophages in infection site. Following laser activation, the greatest F4/80 fluorescence was observed in the MP-MENP + Laser group. This may be due to the superior pro-migration activity of MP-MENP, as well as its polarization capacity on M1 macrophages which can considerably release inflammatory cytokines to recruit the circulating macrophages to infected location. In addition, the potent antibacterial activity of MP-MENP + Laser group may provoke ICD-induced antibacterial immune response, alleviate the immunosuppressive infection microenvironment, and thus facilitate macrophage infiltration. Furthermore, the levels of inflammatory factors secreted by macrophages were quantify using ELISA method, and greater expressions of M1-associated pro-inflammatory cytokines (e.g. TNF-α, IL-1β, IL-6 and IL-12) were detected in MP-MENP and MP-MENP + Laser groups, while the M2-associated anti-inflammatory cytokines (IL-10) showed a reduced secretion (Fig. 5C). Such change in inflammatory factors is promising to elicit immunogenicity and activate effector T cells. Moreover, the ICD-induced antibacterial immune activation was also explored in vivo. After different treatments, HSP70 expression was assessed through IF staining on day 2. According to Fig. 5D and S6, the most effective HSP70 expression was observed in the MP-MENP + Laser group, which suggests a superiority of MP-MENP-mediated PTT in inducing ICD via the enhanced expression of DAMPs. Then the activation of a T cell-dependent antibacterial immune response was estimated. As expected, mice treated with MP-MENP plus laser irradiation exhibited the highest infiltration of effector T helper cells (CD4) and cytotoxic T lymphocyte (CD8) at the site of bacterial infection (Fig. 5D, S7, and S8). The combination of MP-MENP induced infection-associated macrophages repolarization and PTT mediated ICD was highly capable in serving as an immunity amplifier to activate the prosperity of antibacterial immune response, and finally completely eradicated bacterial infection and inhibited its recurrence.

To elucidate the underlying therapeutic mechanism of MP-MENP-mediated synergistic effect of PTT and immunotherapy, MRSA-infected skin tissues were collected to analyze differential gene expression pathway changes between MP-MENP + Laser group and saline group through the whole transcriptome RNA sequencing technique. Figure 5E indicated a uniform distribution of gene expression in different samples from the two groups. Then, differential expression analysis identified 1656 significantly dysfunctional genes (P < 0.05 and |log2(fold change)| ≥ 1), where the expression of 1328 genes was upregulated and 328 genes downregulated in MP-MENP + Laser group compared with saline group (Fig. 5F). The heat map showing differential clustering of related genes (Fig. 5G) also exhibited a wide range of gene expression differences, revealing the robust transcriptome reprogramming in response synergistic therapy mediated by MP-MENP. To understand the pathophysiological relevance to MP-MENP-mediated differentially expressed genes (DEGs), these DEGs were subjected to Gene Ontology (GO) and Kyoto Encyclopedia of Genes and Genomes (KEGG) pathway analysis (Fig. 5H, I). Interestingly, biological processes (BP) enrichment analysis carried out by ClueGO showed that MP-MENP plus laser treatment was involved in leukocyte migration, regulation of cell-cell adhesion, positive regulation of cytokine production, positive regulation of cell adhesion, and positive regulation of response to external stimulus, which are strongly associated with inflammatory and immune response. Subsequently, KEGG pathway enrichment analysis was performed to illustrate the potential signaling pathways of MP-MENP-mediated synergistic therapy. As presented in Fig. 5I, the mechanism of photo-immune treatment by MP-MENP was mainly dependent on PI3K-Akt signaling pathway, cytokine-cytokine receptor interaction, chemokine signaling pathway, MAPK signaling pathway, and T cell receptor signaling pathway. Among them, the PI3K-Akt and MAPK signaling pathway are closely related to macrophage polarization from M2 to M1 phenotype [32, 33]. The significant upregulation of T cell receptor signaling pathway was consistent with the results observed IF assay, revealing a robust immune activation on T cells by MP-MENP-mediated synergistic treatment. Other immune processes, like C-type lectin receptor signaling pathway that involves antigen presentation and the induction of appropriate adaptive immune responses [34], were also positively affected. Moreover, genes related to ABC transporters were found to be enriched, which play a key role in modulating HSP70 expression. Taken together, MP-MENP under laser irradiation could efficiently relieve the immunosuppressive infection microenvironment through inducing macrophage polarization, triggering ICD effect, and activating antibacterial immunity (Fig. 5J).

Further safety examination suggested that the MP-MENP had good hemocompatibility (Figure S9), and were nontoxic to human umbilical vein endothelial cells (HUVEC), RAW264.7 murine macrophage cells, and human hepatic cells LO2 (Figure S10). The histopathological evaluation also confirmed the good safety of MP-MENP without serious damage on the normal anatomical structures of organs (Figure S11).

## Conclusion

In this work, we reported an endogenous cell hitchhiking nano-Trojan Horse for precise visualization and photo-immunotherapy of MDR bacterial infection. This novel-acting MP-MENP nanotheranostic was facilely engineered by mannose-decorated manganese-eumelanin coordination nanoparticles, and shows four primary advantages as follows: (i) two-step monocyte-targeting strategy, a potent synergism of circulating monocyte hitchhiking and the infection-infiltrated monocyte recognition; (ii) dual-modal clinically available imaging, allowing in situ, real-time, noninvasive diagnosis of bacterial infection; (iii) dual-pronged antibacterial capability that attacks both extracellular and intracellular pathogens; (iv) risk-averse photothermal-immunotherapeutic method, which avoids the limitations of antibiotic resistance. With the aid of PTT-mediated ICD and macrophage repolarization by MP-MENP, the antibacterial immunity was successfully activated, accomplishing a highly potent eradication of MRSA infection. Further coupled with great biosafety, the antibiotic-free MP-MENP could represent a promising nanoplatform to alleviate the clinical predicament of infection diagnosis, improve the therapeutic outcomes of MDR bacterial infection, and even potentially shift the current paradigm of personalized antimicrobial stewardship.

### Electronic supplementary material

Below is the link to the electronic supplementary material.

Additional file 1: **Figure S1**. The bacterial viability of (A) MDR *Bacillus*, (B) ESBL-producing *E. coli*, (C) MDR *K. pneumoniae*, and (D) MDR *P. aeruginosa* versus the MP-MENP concentrations with/without laser irradiation (808 nm, 2 W/cm2, 5 min). **Figure S2.** The temperature change at MRSA-infected site from mice treated with saline or MP-MENP, followed by laser irradiation (808 nm, 2 W/cm^2^, 5 min). Figure S3. Representative photographs of the MRSA-infected area within 12 days postinjection in four different treatment groups. **Figure S4.** Semi-quantitative analysis of the average optical density (AOD) of M1 versus M2. M1 AOD = CD86 optical density/F4/80 optical density, M2 AOD = CD206 optical density/F4/80 optical density. **Figure S5.** Semi-quantitative analysis of the relative expression of HSP70 in different groups. **Figure S6.** Semi-quantitative analysis of the AOD of CD8^+^ T cells. CD8^+^ AOD = CD8^+^ optical density/DAPI optical density. **Figure S7.** Semi-quantitative analysis of the AOD of CD4 + T cells. CD4 + AOD = CD4 + optical density/DAPI optical density. **Figure S8.** Quantitative results and photographs of hemolysis activity of MP-MENP with different concentrations. **Figure S9.** Cell viability of HUVEC cells, RAW264.7 cells, and LO2 cells treated with MP-MENP at different concentrations. **Figure S10**. Histopathologic examination of the major organs including heart, liver, spleen, lung, and kidney from mice with saline or MP-MENP injection.

## Data Availability

Data will be available on reasonable request.
