# Supplementary material for: Nanotheranostic Trojan Horse for visualization and photo-immunotherapy of multidrug-resistant bacterial infection
Source: J Nanobiotechnology. 2023 Dec 20;21:492. doi: 10.1186/s12951-023-02267-6 (PMC10731858; doi:10.1186/s12951-023-02267-6)
Supplement: Supplementary file 1 — Additional file 1: Figure S1. The bacterial viability of (A) MDR Bacillus, (B) ESBL-producing E. coli, (C) MDR K. pneumoniae, and (D) MDR P. aeruginosa versus the MP-MENP concentrations with/without laser irradiation (808 nm, 2 W/cm2, 5 min). Figure S2. The temperature change at MRSA-infected site from mice treated with saline or MP-MENP, followed by laser irradiation (808 nm, 2 W/cm2, 5 min). Figure S3. Representative photographs of the MRSA-infected area within 12 days postinjection in four different treatment groups. Figure S4. Semi-quantitative analysis of the average optical density (AOD) of M1 versus M2. M1 AOD = CD86 optical density/F4/80 optical density, M2 AOD = CD206 optical density/F4/80 optical density. Figure S5. Semi-quantitative analysis of the relative expression of HSP70 in different groups. Figure S6. Semi-quantitative analysis of the AOD of CD8+ T cells. CD8+ AOD = CD8+ optical density/DAPI optical density. Figure S7. Semi-quantitative analysis of the AOD of CD4 + T cells. CD4 + AOD = CD4 + optical density/DAPI optical density. Figure S8. Quantitative results and photographs of hemolysis activity of MP-MENP with different concentrations. Figure S9. Cell viability of HUVEC cells, RAW264.7 cells, and LO2 cells treated with MP-MENP at different concentrations. Figure S10. Histopathologic examination of the major organs including heart, liver, spleen, lung, and kidney from mice with saline or MP-MENP injection. [file 12951_2023_2267_MOESM1_ESM.docx]

**Article type: Research Paper**

**Nanotheranostic Trojan Horse for Visualization and Photo-Immunotherapy of Multidrug-Resistant Bacterial Infection**

Xin Pang^1,#,🖂^, Haohang Xu^1,#^, Qishun Geng^2^, Heng Liu^3^, Xiao Zhang^1^, Mingsan Miao^4,🖂^

1. School of Pharmacy, Henan University of Traditional Chinese Medicine, Zhengzhou 450046, China.
2. China-Japan Friendship Hospital (Institute of Clinical Medical Sciences), Chinese Academy of Medical Sciences & Peking Union Medical College, Beijing 100193, China.
3. Department of Radiology, PLA Rocket Force Characteristic Medical Center, Beijing 100088, China
4. Academy of Chinese Medical Sciences, Henan University of Traditional Chinese Medicine, Zhengzhou 450046, China

#These two authors contributed equally to this work.

🖂 Corresponding author: E-mails: pangxin116@163.com (X. Pang), miaomingsan@163.com (M. Miao)


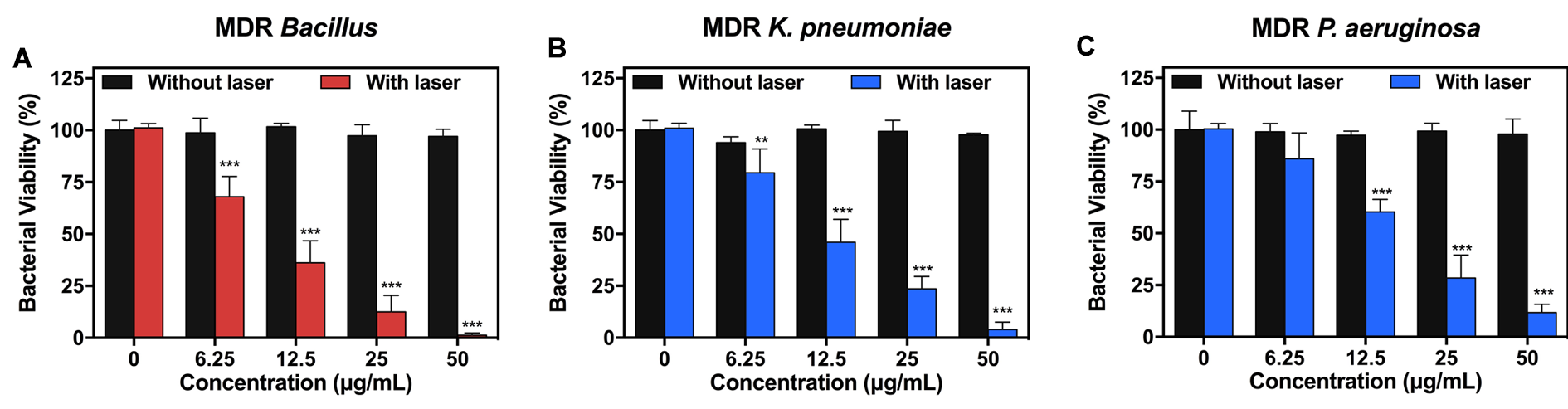


**Figure S1.** The bacterial viability of (A) MDR Bacillus, (B) MDR *K. pneumoniae,* and (C) MDR *P. aeruginosa* *versus* the MP-MENP concentrations with/without laser irradiation (808 nm, 2 W/cm^2^, 5 min).


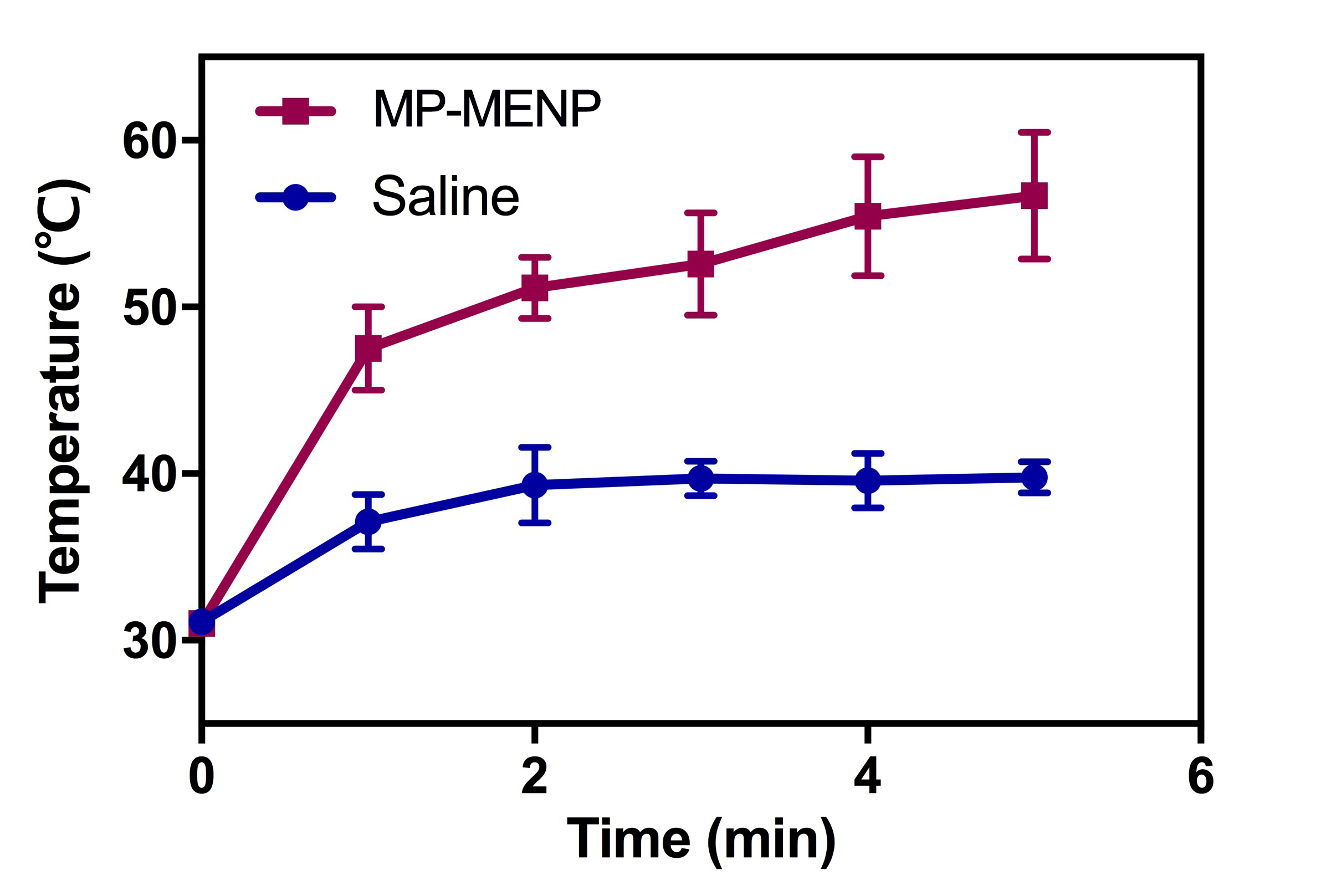


**Figure S2.** The temperature change at MRSA-infected site from mice treated with saline or MP-MENP, followed by laser irradiation (808 nm, 2 W/cm^2^, 5 min).


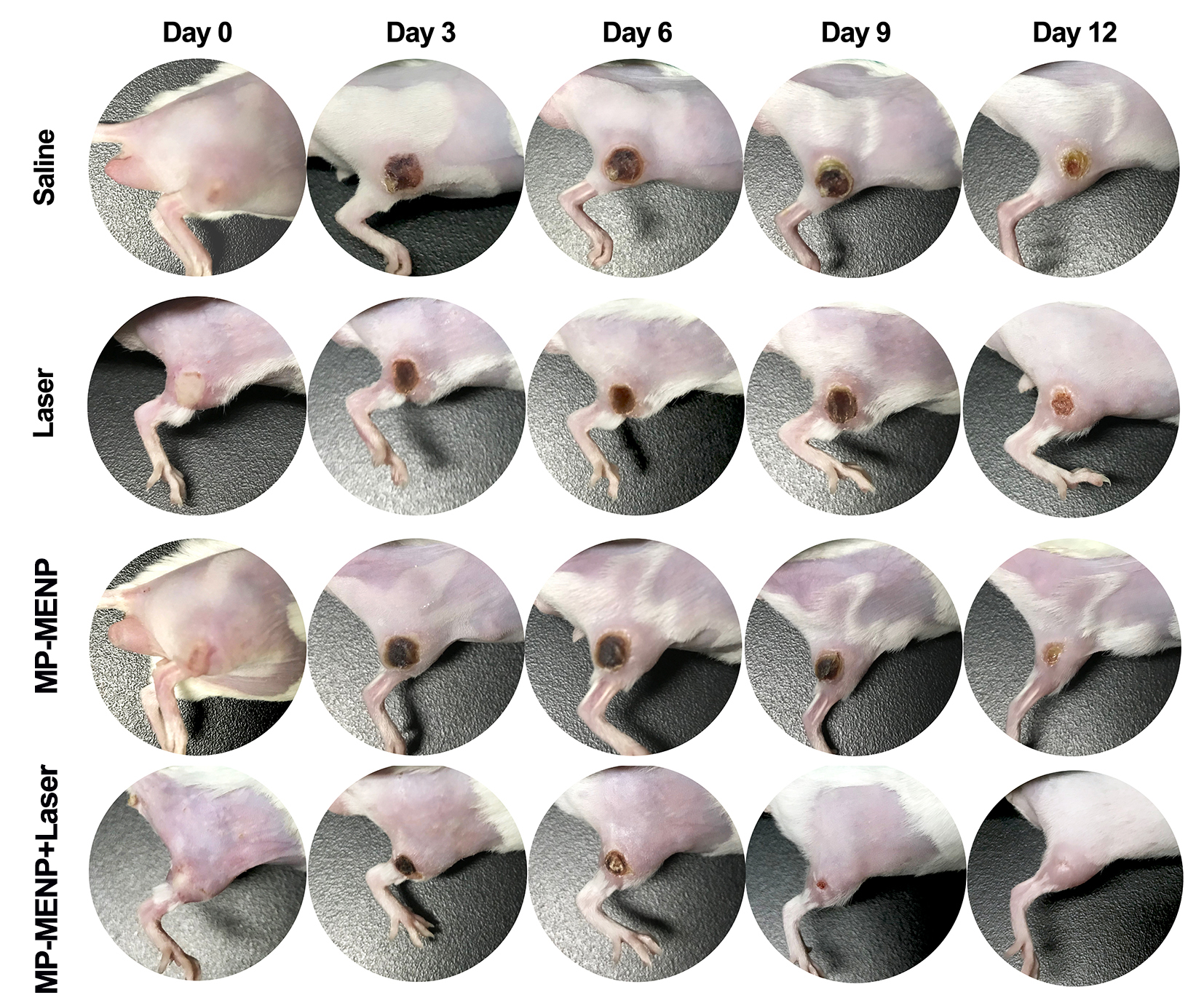


**Figure S3.** Representative photographs of the MRSA-infected area within 12 days postinjection in four different treatment groups.


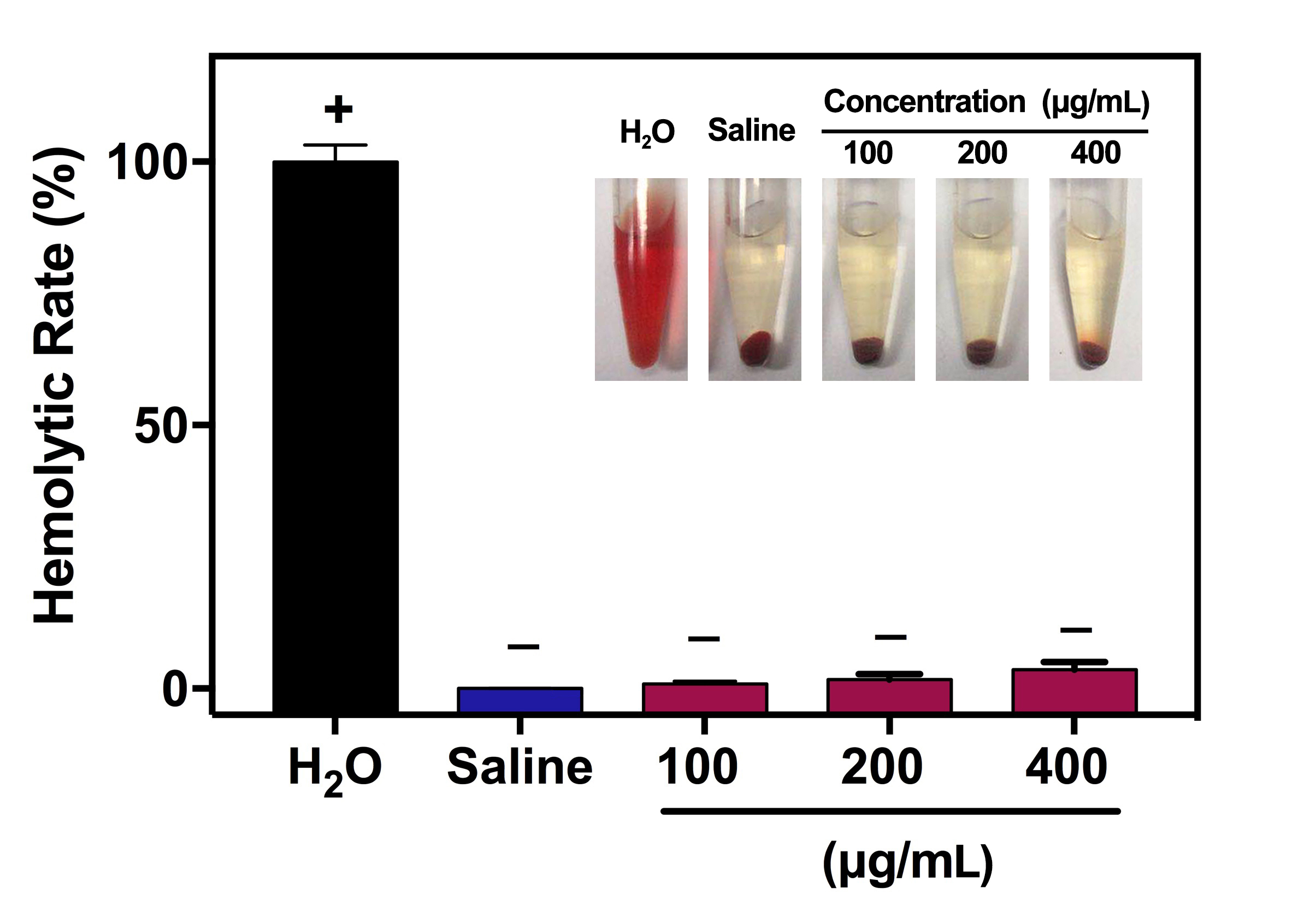


**Figure S4.** Quantitative results and photographs of hemolysis activity of MP-MENP with different concentrations.


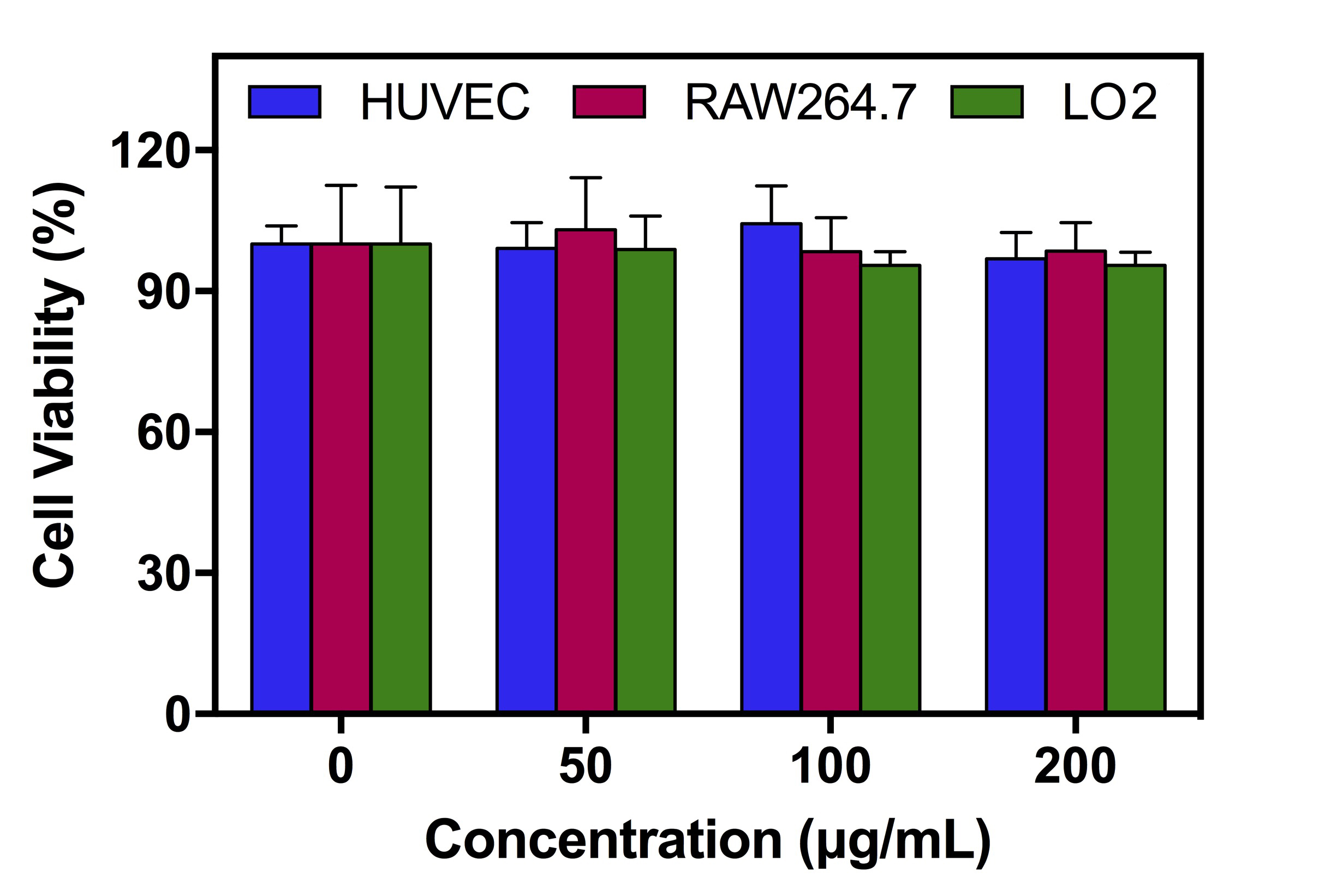


**Figure S5.** Cell viability of HUVEC cells, RAW264.7 cells, and LO2 cells treated with MP-MENP at different concentrations.


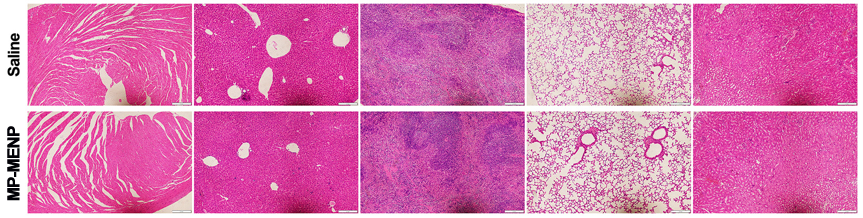


**Figure S6.** Histopathologic examination of the major organs including heart, liver, spleen, lung, and kidney from mice with saline or MP-MENP injection.
